# A comparative ultrastructure study of the tardigrade *Ramazzottius varieornatus* in the hydrated state, after desiccation and during the process of rehydration

**DOI:** 10.1371/journal.pone.0302552

**Published:** 2024-06-06

**Authors:** Simon Galas, Emilie Le Goff, Chantal Cazevieille, Akihiro Tanaka, Pierre Cuq, Stephen Baghdiguian, Takekazu Kunieda, Nelly Godefroy, Myriam Richaud

**Affiliations:** 1 IBMM, University of Montpellier, CNRS, ENSCM, Montpellier, France; 2 ISEM, University of Montpellier, CNRS, IRD, Montpellier, France; 3 INM, University of Montpellier, INSERM, Montpellier, France; 4 Department of Biological Sciences, Graduate School of Science, The University of Tokyo, Tokyo, Japan; UMinho CBMA: Universidade do Minho Centro de Biologia Molecular e Ambiental, PORTUGAL

## Abstract

Tardigrades can survive hostile environments such as desiccation by adopting a state of anhydrobiosis. Numerous tardigrade species have been described thus far, and recent genome and transcriptome analyses revealed that several distinct strategies were employed to cope with harsh environments depending on the evolutionary lineages. Detailed analyses at the cellular and subcellular levels are essential to complete these data. In this work, we analyzed a tardigrade species that can withstand rapid dehydration, *Ramazzottius varieornatus*. Surprisingly, we noted an absence of the anhydrobiotic-specific extracellular structure previously described for the *Hypsibius exemplaris* species. Both *Ramazzottius varieornatus* and *Hypsibius exemplaris* belong to the same evolutionary class of Eutardigrada. Nevertheless, our observations reveal discrepancies in the anhydrobiotic structures correlated with the variation in the anhydrobiotic mechanisms.

## 1. Introduction

Tardigrades are tiny metazoan animals that range in size from approximately 0.1–1.2 mm and have four pairs of legs [1]. They can be called “water bears” because of their appearance and “moss piglets” because of where they can be found. Nearly 1500 tardigrades species have thus far been described [2], which are distributed from the depths of the oceans to the highest mountain peaks [3]. The worldwide distribution of tardigrade species can be either endemic or cosmopolitan [4–8], and their transport by birds or snails has recently been suggested [9–11].

As earlier as their discovery in the 18^th^ century [12], tardigrades have demonstrated an ability to adopt a latent state due to a shortage of water, which is called anhydrobiosis. These tardigrades, rather terrestrial species, can enter an anhydrobiosis state in response to desiccation to form an anhydrobiote, allowing the organism to wait for the return of water [13, 14]. Thus, by reaching nearly complete desiccation, tardigrades can survive for many years as anhydrobiotes [15–18]. During the course of desiccation, tardigrades contract and retract their whole body to assume a characteristic “tun”-shaped anhydrobiote structure. Tardigrades can then lose up to 97% of their bound and free body water content [19].

Other invertebrates, such as rotifers, nematodes and dipteran larvae [12, 20–23], can enter anhydrobiosis and some of them are also tolerant to other extreme physical stresses. For example, the nematode *Panagrolaimus superbus* displays tolerance to ultra-low temperature (-196°C), X-radiations (500Gy) or ultracentrifugation (400,000xg) [24] and bdelloids rotifers are able to withstand high doses of ionizing radiation, up to 1000 Gy [25, 26]. Likewise, *Adineta vaga* is known for its resistance to X-ray, protons and Fe ions [27]. However the particularity of the tardigrade is that it resists a more extensive set of stresses. Indeed, tardigrades are resistant to temperatures ranging from -272 to +150°C [28, 29], very high pressures (up to 7.5 Gpa) equivalent to that at a depth of up to 180 km from the Earth’s surface [30], radiation at levels up to 5000 Gy [31–33] and exposure to solar radiation at a low Earth orbit in a space vacuum during a ten-day space flight [34]. These characteristics make them an emerging model for space biology [35].

To date, the genomes of four tardigrade species are available [36]. The genomes of two Eutardigrada species: *Ramazzottius varieornatus* (*Ram*. *varieornatus*) and *Hypsibius exemplaris (Hys*. *exemplaris)* [37–39], enabled the identification of gene products involved in anhydrobiosis. For instance, the Dsup (damage suppressor) gene was identified in *Ram*. *Varieornatus* and was suggested to protect both human and plant cells from gamma ray irradiation [31, 38, 40] as well as human cultured cells from oxidation by free radicals [31, 38]. The molecular capacity of the Dsup gene products to protect nucleosomes from direct oxidation by hydroxyl radicals was thereafter evidenced by an *in vitro* assay [41].

While *Ram*. *varieornatus* is tolerant to a rapid desiccation process (minutes), *Hys*. *exemplaris* can undergo effective anhydrobiosis after only an obligate preconditioning period (hours) [14, 36, 42].

Accordingly, both species show contrasting gene expression in response to desiccation [36, 43]. Thus, while *Ram*. *varieornatus* is believed to express anhydrobiosis involved genes constitutively, *Hys*. *exemplaris* requires a *de novo* expression induction of orthologs genes [36]. To date, tardigrade species belonging to the Heterotardigrada class seem to lack *bona fide* Dsup orthologs [41].

It has been shown [44] that tardigrades belonging to the Eutardigrada class, such as *Ram*. *varieornatus* and *Hys*. *exemplaris*, also possess genes [38, 45, 46] encoding proteins that are involved in resistance to desiccation stress, such as the cytosolic abundant heat-soluble (CAHS), secretory abundant heat-soluble (SAHS), late embryogenesis abundant mitochondrial (RvLEAM), and mitochondrial abundant heat-soluble (MAHS) proteins. These intrinsically disordered proteins (IDPs) are involved in the maintenance of cellular structures during desiccation processes [38, 46–51].

Species of the Eutardigrada possess specific genes involved in stress resistance that differ from those of Heterotardigrada, but some discrepancies have also been reported among eutardigrade species. For example, *Ram*. *varieornatus* possesses a trehalose-6-phosphate synthase gene, while *Hys*. *exemplaris* does not [43]. Trehalose-6-phosphate synthase can produce the nonreducing sugar trehalose [52], which has been proposed to play a role in mediating desiccation tolerance in some organisms, such as *Caenorhabditis elegans* (Maupas, 1899) [53], *Saccharomyces cerevisiae* (Meyen, 1838) [54] and chironomids, by vitrifying their cellular contents. However, other desiccation-tolerant invertebrates, such as rotifers, do not require this sugar [55–58] and the presence of the trehalose is still unclear in tardigrades [59–63].

To date, few reports have attempted to describe the ultrastructures of anhydrobiotic organisms [64–66]. Halberg *et al*. [67] described the tun morphology of the *Richtersius coronifer* with an emphasis on muscular organization, while Czernekova *et al*. [68, 69] investigated the internal morphologies of dehydrated organs, tissues and cells in the same species. Poprawa *et al*. [14] characterized ultrastructure of storage cells in tuns of *Hys*. *exemplaris*.

In a previous report [70], we used electron microscopy to compare hydrated specimens and anhydrobiotic tuns of *Hys*. *exemplaris*. We highlighted deep modifications occurring up to the subcellular level in the anhydrobiote and during the course of exit from anhydrobiosis. We also uncovered the materialization of an anhydrobiote-specific and reversible extracellular structure [70].

In the present study, we studied the structures and ultrastructures of the cells and organelles of anhydrobiotic *Ram*. *varieornatus* specimens by electron microscopy and compared them to the ultrastructures of active hydrated specimens. Finally, we compared strategies used by the Eutardigrada species *Ram*. *varieornatus* and *Hys*. *exemplaris* to resist anhydrobiosis.

## 2. Materials and methods

### 2.1 Materials

The Yokozuna-1 strain of the extremotolerant *Ram*. *varieornatus (*Bertolani and Kinchin, 1993) [71], (Eutardigrada, Hypsibiidae), provided by Takekazu Kunieda (University of Tokyo), was used for all experiments. Tardigrades were cultured as previously described [29]. They were fed with the unicellular algae *Chlorella vulgaris* (Beijerinck, 1890) [72] *(*strain A60*)* on 2% Bacto agar plates prepared with Volvic water and incubated at 20°C under constant dark conditions. Algae were purchased from the Sciento Company (Manchester, UK).

### 2.2 Desiccation protocol

Twenty specimens in a drop of water were placed on a filter paper inside Petri dish, which were left at room temperature (20–22°C) and relative humidity (RH) (between 30–36%) for one hour. To confirm good dehydration, the dessication process of the specimens was monitored by direct observation under a stereomicroscope in order to assure that the tardigrades underwent a proper anhydrobiosis process and formed tuns. We therefore control size reduction and the total absence of movement. The anhydrobionts were stored at 20°C and at room RH (between 30–36%) in an incubator for one week before analysis.

### 2.3 Rehydration protocol

To rehydrate the desiccated *Ram*. *varieornatus* after one week of dehydration, Volvic water droplets were added to the filters. Tardigrades were maintained in water at room temperature (20–22°C) and prepared for TEM after 5 and 15 minutes of contact with liquid. Specimens of *Ram*. *varieornatus* start to move around 5 minutes after rehydration.

### 2.4 Transmission electron microscopy

According to Richaud et al. [70], samples were fixed in 2.5% glutaraldehyde in PHEM buffer (1X, pH 7.4) overnight at 4°C, rinsed in PHEM buffer and postfixed in 0.5% osmic acid for 2 hours in the dark at room temperature. After two rinses in PHEM buffer, samples were dehydrated in a graded series of ethanol (30–100%) and embedded in EmBed 812 using an automated microwave tissue processor for electronic microscopy (Leica EM AMW). Ultrathin sections (70 nm; Leica-Reichert Ultracut E) were collected from different levels of each block, counterstained with 1.5% uranyl acetate in 70% ethanol and lead citrate and observed using a Tecnai F20 transmission electron microscope at 200 kV at the CoMET MRI facilities (INM, Montpellier, France). For TEM, five tardigrades were analyzed for each condition: tuns, rehydrated for 5 minutes, rehydrated for 15 minutes and hydrated.

### 2.5 Tardigrade size

We measured the sizes of hydrated and tun tardigrades from the tip of the head to the extreme end of the body without legs. Measurements from DIC images obtained using a Zeiss LSM880 Fast Airyscan confocal microscope at the DBS-Optique MRI facilities (Montpellier, France) were determined with ImageJ software. For each condition, five specimens were measured.

### 2.6 Number of nuclei

We counted the number of nuclei in hydrated and tuned animals. Tardigrades were fixed for 30 min with 4% paraformaldehyde (PFA) in mineral water (Volvic) and then permeabilized for 20 min at room temperature with 0.2% Triton X-100 in PBS buffer (Amresco, Inc.). Fixed and permeabilized tardigrades were labeled with DAPI (Euromedex 1050-A) at 1 μg/mL in PBS for 30 min at room temperature. Finally, the specimens were rinsed several times with PBS and mounted in Dako. Counts from DAPI z-stack stained images obtained using a Zeiss LSM880 Fast Airyscan confocal microscope at the DBS-Optique MRI facilities (Montpellier, France) were determined with ImageJ software. For each condition, hydrated and dehydrated tardigrades, the number of nuclei was counted in five animals, *i*.*e*. 10 tardigrades in total.

### 2.7 Mitochondrial size

We measured the mitochondrial sizes under every condition: tuns, rehydrated for 5 minutes, rehydrated for 15 minutes and hydrated. For each condition, mitochondria were observed in each cell types and in the same proportions to avoid sampling bias. The sizes of 150, 110, 136 and 140 mitochondria were measured in each group, respectively, using ImageJ software. Mitochondria were measured in cross-sections through and through on the longer side.

### 2.8 Statistical analysis

According to Richaud et al. [70], we used XLSTAT software (Addinsoft, New York, NY, USA) to compare mitochondrial sizes among animals that were dehydrated, rehydrated for 5 or 15 minutes and hydrated and to compare the body sizes and numbers of nuclei between hydrated and dehydrated animals.

## 3. Results

### 3.1. Comparison of hydrated and anhydrobiotic Ramazzottius varieornatus

#### 3.1.1. Cell compaction of anhydrobiotic tardigrades

**Fig 1:**
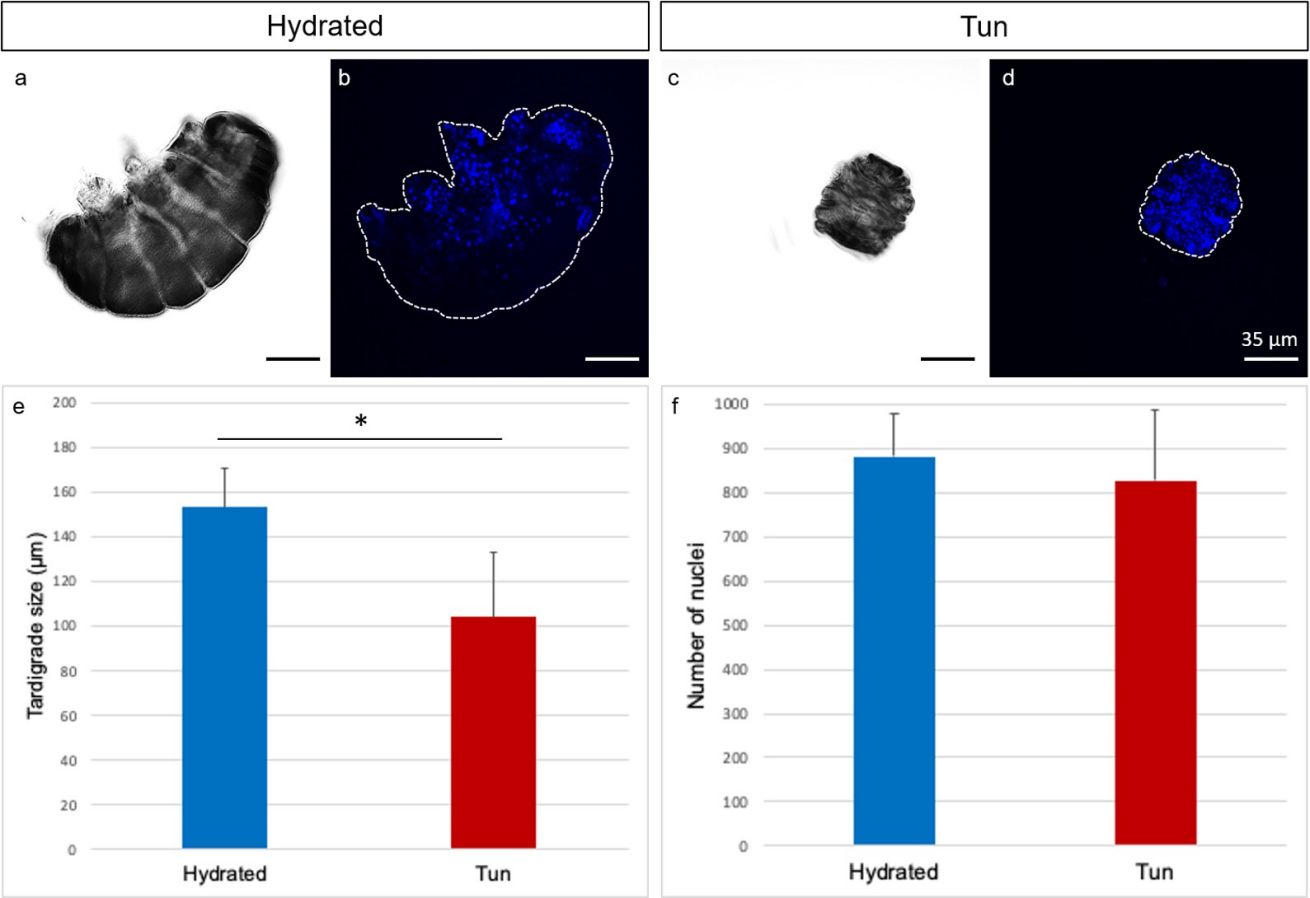
Comparison between hydrated and anhydrobiotic *Ram*. *varieornatus*: (a-d) Confocal microscopy images with DIC (a and c) and DAPI (b and d) staining. (e-f) Statistics on body sizes and nucleus numbers. (e) Error bars indicate the standard deviation and the star indicates a significant difference (Kolmogorov-Smirnov test, p = 0.048; α = 0.05). (f) Error bars indicate the standard deviation.

We first used confocal laser microscopy with differential interference contrast (DIC) to obtain a global view of the external morphologies of hydrated specimens versus anhydrobiotic tuns. In parallel, we used the DNA-specific dye DAPI to enable nuclei counting. Fig 1A and 1C shows representative images of the characteristic contraction in anhydrobiotic tun compared to hydrated specimen. Length measurements of hydrated specimens revealed an average size of 153 +/- 17 μm, while the anhydrobiotic tuns showed an average size of 104 +/- 29 μm (Fig 1E), revealing a size reduction of 32% (Fig 1E). However, staining the nuclei of both the hydrated (Fig 1B) and anhydrobiotic groups (Fig 1D) with DAPI revealed a nonsignificant t difference in the total cell counts (Fig 1F).

#### 3.1.2. Comparative analysis of cell structure and ultrastructure

**Fig 2:**
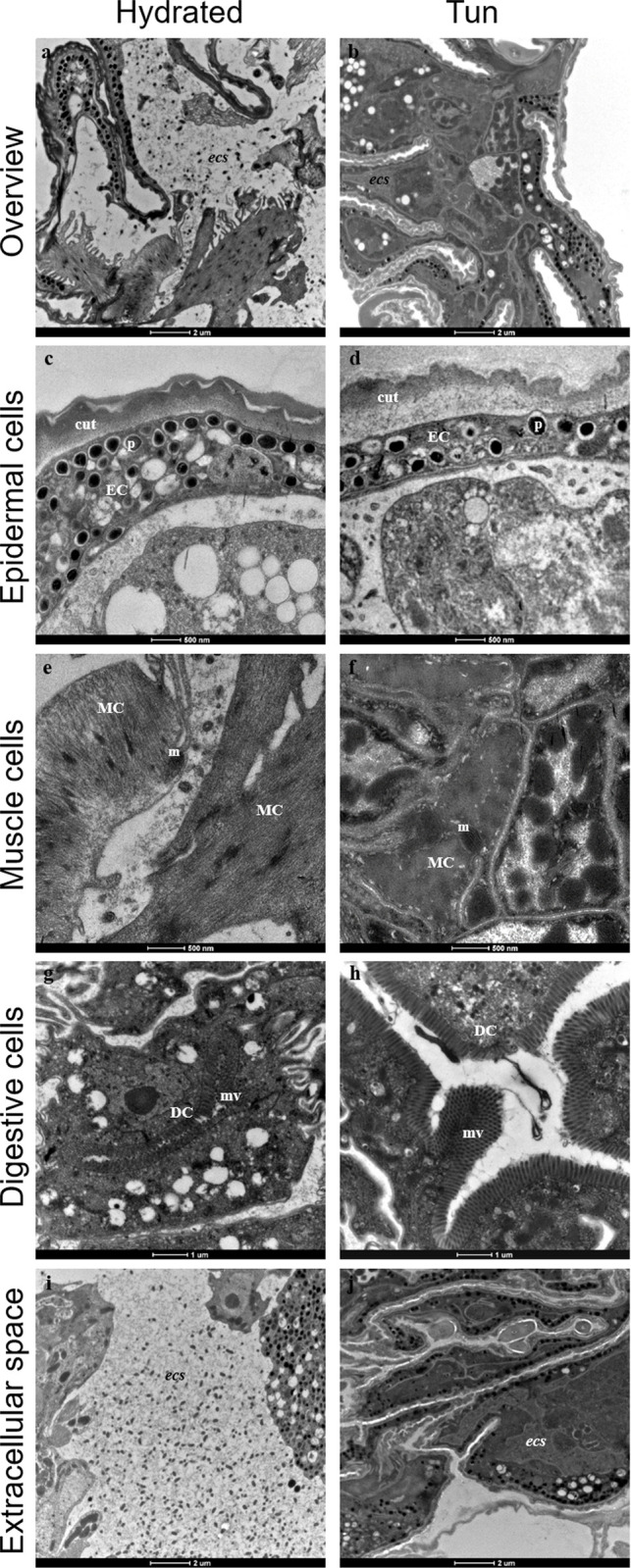
Ultrastructures of *Ram*. *varieornatus* under the hydrated and tun states. (**a**, **b**) Overview of a body part. (**c**, **d**) Ultrastructure of epidermal cells. (**e**, **f**) Ultrastructure of muscle cells. (**g**, **h**) Ultrastructure of digestive cells. (**i**, **j**) Extracellular space overview. Cut: cuticle, DC: digestive cell, EC: epidermal cell, MC: muscle cell, m: mitochondria, mv: microvilli, p: pigment, *ecs*: extracellular space.

Hydrated individuals showed a large space between cells, named extracellular space (*ecs*) (Fig 2A). Sizes of this space throughout the body of the tardigrade were not similar. Epidermal cells, bordered by the cuticle, were clearly visible together with numerous pigmented vesicles (Fig 2C). Muscle cells possessed long fibers with long dark mitochondria (Fig 2E), and digestive cells appeared with long villosities (Fig 2G). Additionally, numerous lipid droplets were observed inside the cells regardless of the cell type.

We also observed *ecs* (Fig 2B) features in the anhydrobiotic tun group (Fig 2B). We observed a space between the epidermal cells and the cuticle (Fig 2D). The global structure of this cell type was not affected by dehydration (Fig 2D) except a cell compaction. Indeed, epidermal cells appear to be thinner in tuns (Fig 2D). This may be due to dehydration-induced compaction. Conversely, muscular fibers were less discernible in the anhydrobiotic tun group than in the hydrated group because of compaction (Fig 2F). Similar to the hydrated tardigrades, the digestive cells of the anhydrobiotic group exhibited long villosities (Fig 2H), and lipid droplets were still present. No apoptotic cells were observed in any of the specimens.

In both the hydrated specimens and anhydrobiotic tun groups, dot-like structures were observable in the *ecs* (Fig 2I and 2J).

**Fig 3:**
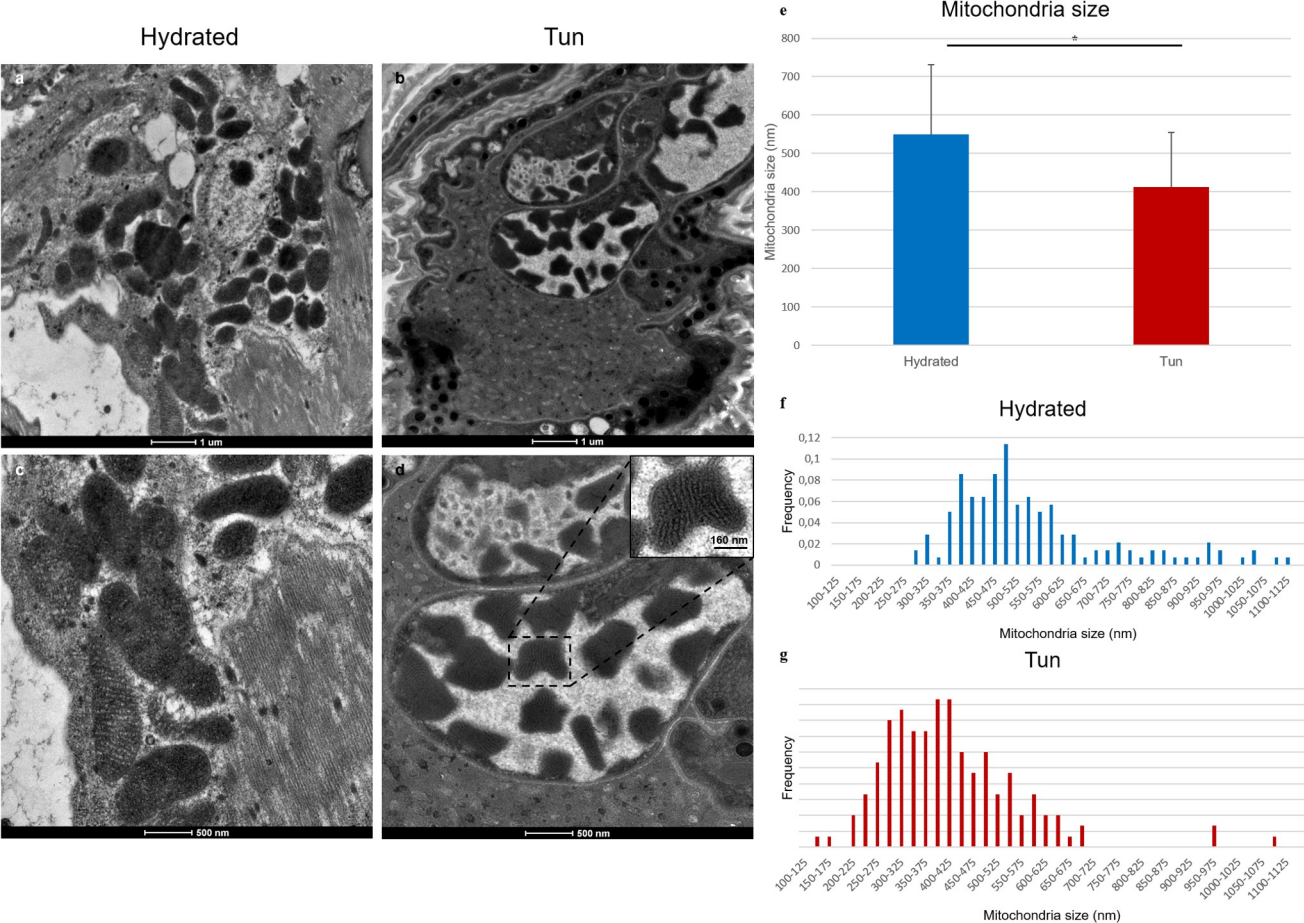
Comparison of mitochondria in hydrated and anhydrobiotic *Ram*. *varieornatus* cells. (**a**-**d**) Transmission electron microscopy images. (**e**) Mean mitochondrial size. The error bars indicate the standard deviation, and the stars indicate significant differences (Student’s t-test, α = 0.05). Table 1 shows the complete statistical results. (**f**, **g**) Histogram of the mitochondrial size frequencies of hydrated tardigrades (**f**) and desiccated tardigrades (**g**). m: mitochondria.

The hydrated specimens and anhydrobiotic groups showed comparable organelle structures, with numerous mitochondria being observed in both groups. Moreover, the mitochondrial structures were comparable (Fig 3A and 3C vs 3B and 3D) and not degraded. Surprisingly, the mitochondrial cristae in the anhydrobiotic tun group were comparable to those in hydrated animals (Fig 3C and 3D); however, a statistically significant size reduction of 24% (Fig 3E) was observed in mitochondria of the anhydrobiotic tun group compared with the hydrated specimens group. We observed many mitochondria with atypical shapes in the anhydrobiotic tun group (Fig 3B and 3D) that were not observable in the hydrated specimen group (Fig 3A and 3C).

**Table 1:** Statistical results of the mitochondrial sizes (nm) in cells from *Ram*. *varieornatus* in four stages: tuns (A), after 5 minutes of rehydration (B), after 15 minutes of rehydration (C) and hydrated (D).

Stage	Mean ± SD	Code	p-value*	n
Tun	413 ± 142	A	0.0158 (B)	150
			< 0.0001 (C)	
			< 0.0001 (D)	
Rehydrated for 5 min	453 ± 115	B	0.0158 (A)	110
			< 0.0001 (C)	
			< 0.0001 (D)	
Rehydrated for 15 min	572 ± 136	C	< 0.0001 (A)	136
			< 0.0001 (B)	
			0.2562 (D)	
Hydrated	550 ± 181	D	< 0.0001 (A)	140
			< 0.0001 (B)	
			0.2562 (C)	

*Student’s t-test, ⍺ = 0.05

Fig 3F and 3G shows the size distribution frequencies of up to 140 and 150 mitochondria measured in both the hydrated and anhydrobiotic tun groups respectively.

### 3.2. Temporal change in anhydrobiotic tuns during rehydration

To better understand the functional structures of stress-resistant anhydrobiotic tuns, we assessed the ultrastructural changes in the anhydrobiotic tun group over the course of rehydration. Because anhydrobiotic tuns take only a few minutes (10–20 minutes) to wakeup (size recovery and detectable movements) from dehydration, they were dehydrated for one week, rehydrated for 5 and 15 minutes and then assessed by TEM.

#### 3.2.1. Rehydration of anhydrobiotic tuns for 5 minutes

**Fig 4:**
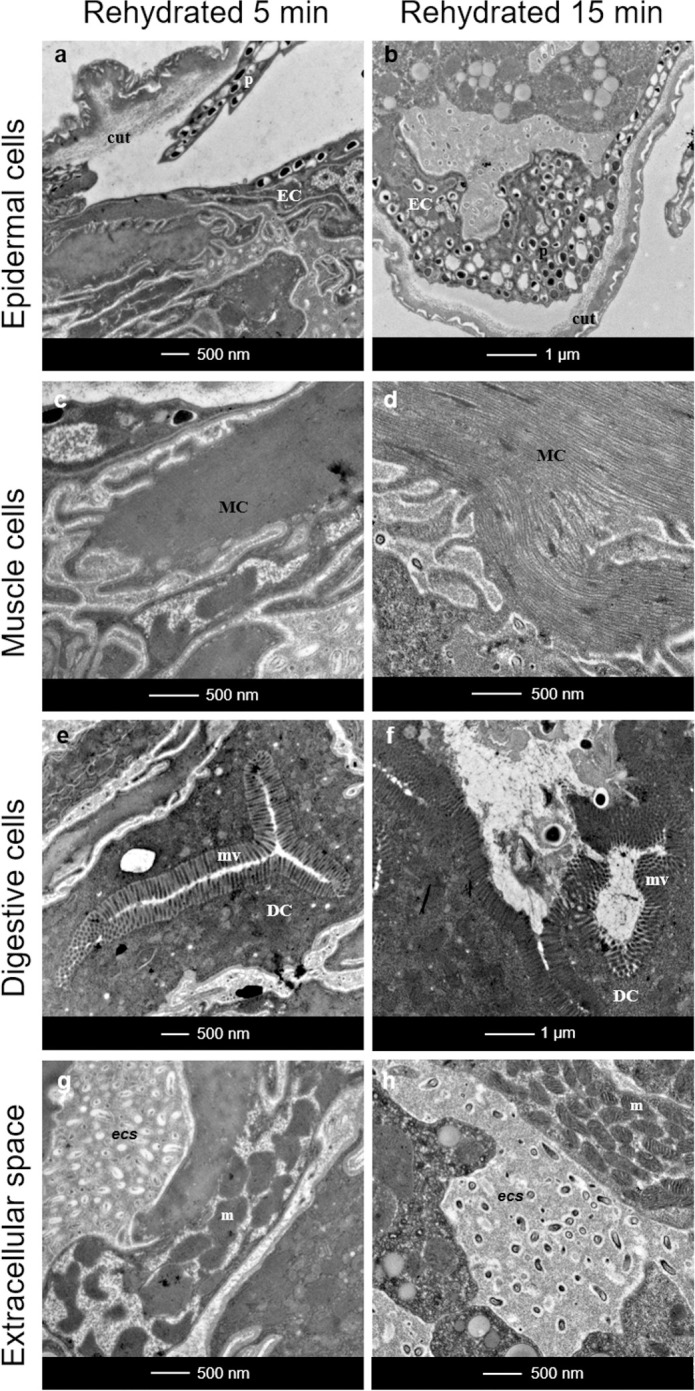
Ultrastructure of *Ram*. *varieornatus* after 5 and 15 minutes of rehydration. (**a**, **b**) Ultrastructure of epidermal cells. (**c**, **d**) Ultrastructure of muscle cells. (**e**, **f**) Ultrastructure of digestive cells. (**g**, **h**) Extracellular space overview. cut: cuticle, DC: digestive cell, EC: epidermal cell, MC: muscle cell, m: mitochondria, mv: microvilli, p: pigment, *ecs*: extracellular space.

**Fig 5:**
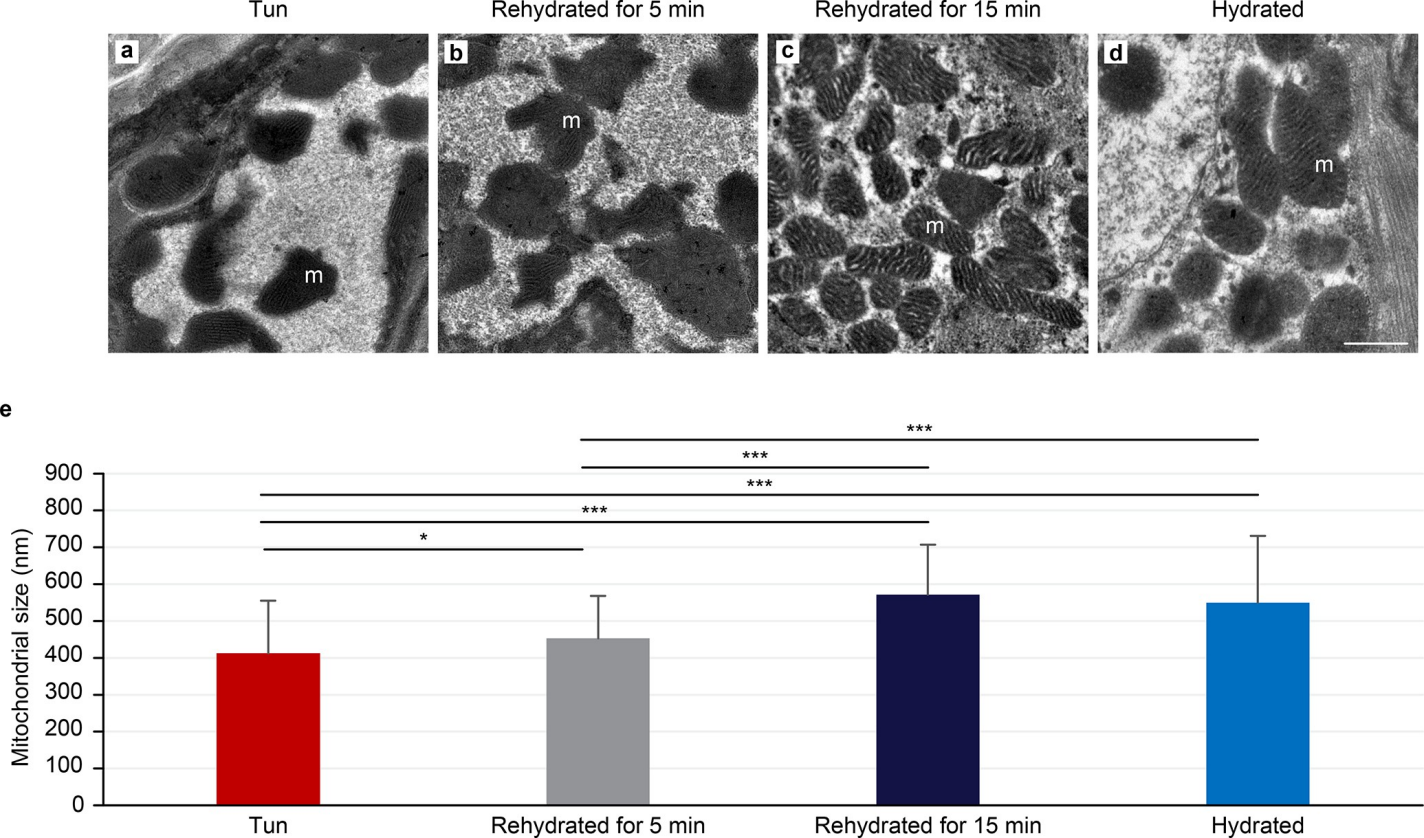
Comparison of mitochondria from *Ram*. *varieornatus* in four stages: Tuns, after 5 minutes of rehydration, after 15 minutes of rehydration and hydrated. (**a**–**d**) Transmission electron microscopy images. Scale bar a-d = 500 nm. (**e**) Mean mitochondrial sizes. The error bars indicate the standard error of the mean. <*> indicates a significant difference at p < 0.05 (Student’s t-test, α = 0.05). <***> indicates a significant difference at p < 0.0001 (Student’s t-test, α = 0.05). See Table 1 for all of the statistical results. m: mitochondria.

After 5 minutes of rehydration, *Ram*. *varieornatus* specimens begin to move and become active. We observed a size evolution that fell between that of the hydrated and anhydrobiotic specimens. Following this observation, we again noticed persistence of the anhydrobiotic state with decoupling between the epidermal cells and the cuticle (Fig 4A). Moreover, the global structure of the epidermal cells containing the already described vesicles was maintained (Fig 4A versus Fig 2C and 2D), and muscle cells exhibited a normal structure with long contractile fibers (Fig 4C). Gut cells also showed a normal ultrastructure compared to those of the hydrated group (Fig 4E versus Fig 2G and 2H). The mitochondrial size was intermediate between those in the anhydrobiotic and hydrated groups of tardigrades (Fig 5B and 5E). Mitochondrial size differences between the anhydrobiotic and hydrated groups were evident based on their size frequency distributions (S1 Fig). More than 110 mitochondria were assessed, and their intermediate sizes were also confirmed by statistical analysis (Table 1). We observed a higher concentration of mitochondria around muscle fibers. Previously described lipid droplets were still observable inside the cells (data not shown) as were the dot-like structures in the *es* (Fig 4G).

#### 3.2.2. Rehydration of anhydrobiotic tuns for 15 minutes

Fifteen minutes after rehydration, *Ram*. *varieornatus* have a normal activity (mobility, food consumption, movements) and are fully active. The tardigrade size was already comparable to that of the hydrated group. In agreement with this observation, the epidermal cells recovered contiguously with the cuticle (Fig 4B), and the muscle cells appeared as classical long fibers, like in the hydrated group (Fig 4D versus Figs 2E, 2F and 4E). In addition, the digestive cells showed a normal structure compared with that in the hydrated group (Fig 4F versus Figs 2G, 2H and 4E). Moreover, the mitochondrial sizes were equivalent to those in the hydrated control group (Fig 5C and 5E). This was shown by evaluating the size frequency distribution of up to 136 mitochondria (S1 Fig) and confirmed by statistical analysis (Table 1). Finally, the lipid droplets, previously described in other conditions were still present, as were the dot-like structures in the *ecs* (Fig 4H).

## 4. Discussion

*Ramazzottius varieornatus* can cope with rapid dehydration and is known to be one of the most resilient to desiccation among the limno-terrestrial tardigrades [36, 38]. However, no information on internal reorganization during anhydrobiosis is available.

We have previously reported [70] that upon desiccation, *Hys*. *exemplaris* shows active secretory cells that are closely related to a specific and reversible extracellular structure surrounding each cell. This specific extracellular structure and the accompanying secretory cells disappear during rehydration, implying their direct association with resistance to dehydration stress. However, *Hys*. *exemplaris* is more sensitive to desiccation than *Ram*. *varieornatus* [38, 43, 44] and thus requires preconditioning steps to achieve successful anhydrobiosis [42, 47, 73].

**Fig 6:**
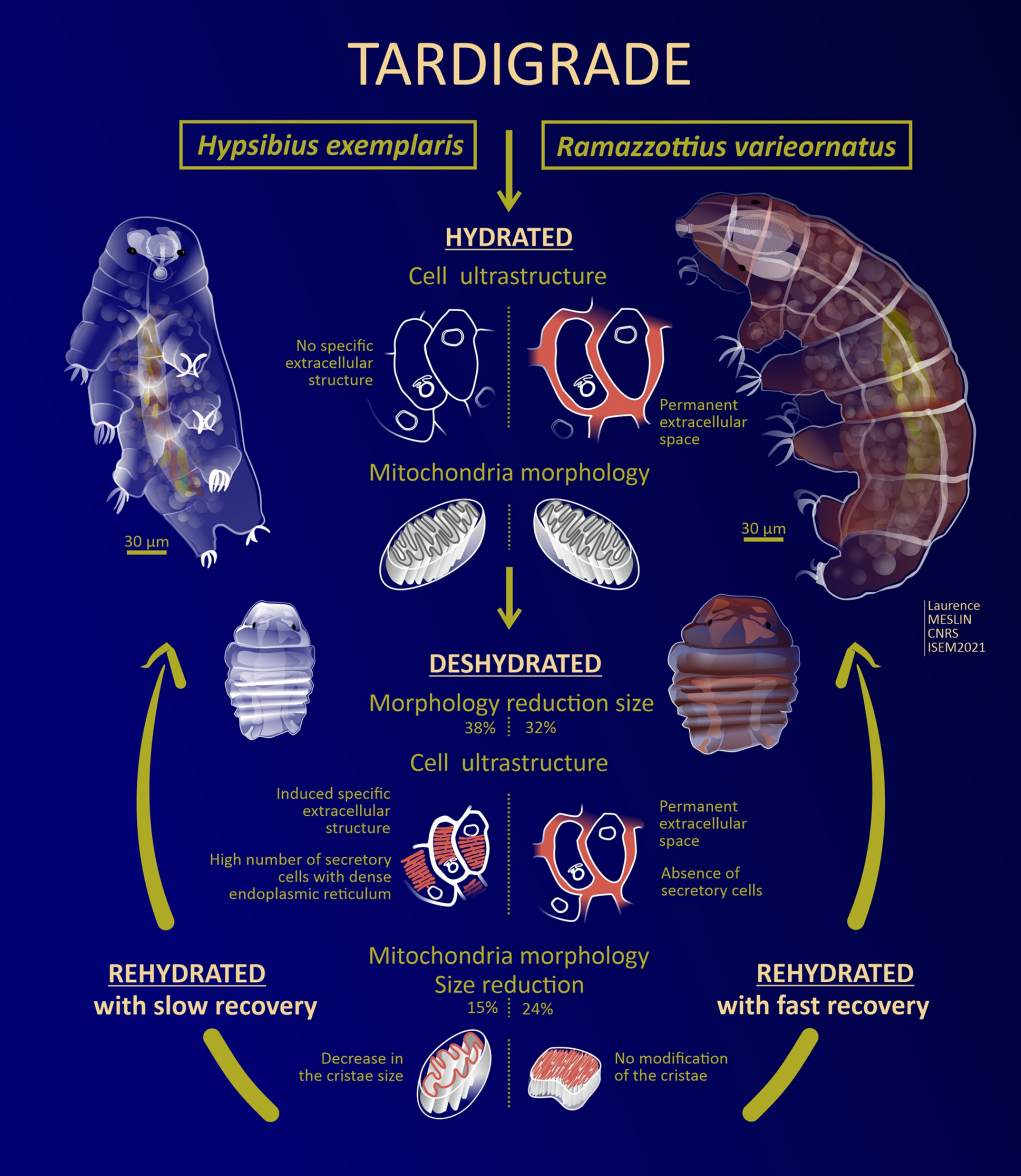
Graphical representation of the ultrastructural divergences between *Ram*. *varieornatus* and *Hys*. *exemplaris* during the dehydration and anhydrobiote formation processes. Abstract design: Laurence Meslin CNRS, ISEM 2023.

Fig 6 summarizes their ultrastructural divergences during the dehydration process and anhydrobiote formation. Contrary to *Hys*. *exemplaris* [70], no secretory cells with a dense network of endocytoplasmic reticulum were found in *Ram*. *varieornatus*.

The presence of active secretory cells during the formation of the *Hys*. *exemplaris* tuns was suggested to be associated with the production of a specific extracellular structure surrounding each cell [70]. In agreement with this observation, we were unable to detect this specific extracellular structure in the tuns (Fig 2) of the *Ram*. *varieornatus*. Moreover, *ecs* is always present in *Ram*. *varieornatus*, anhydrobionts and hydrated specimens, and its pre-existence could explain the high capacity of *Ram*. *varieornatus* to resist anhydrobiosis stress, its speed to reach the tun stage without preconditioning and its speed of return from anhydrobiotic state with an active form.

The shape of mitochondria in *Ram*. *varieornatus* and *Hys*. *exemplaris* tuns cells represents another divergent ultrastructural characteristic between the two species (Fig 5). Compared to those in the hydrated groups, the anhydrobiotic mitochondria of *Hys*. *exemplaris* exhibited a reduced size (15%) and a decreased cristae size [70], while those of *Ram*. *varieornatus* showed a slightly greater size reduction (24%) but comparable cristae (Fig 5A versus 5D). This cristae size difference between *Ram*. *varieornatus* and *Hys*. *exemplaris* may explain the respiration reactivation and faster anhydrobiotic exit of *Ram*. *varieornatus* anhydrobionts.

In the present study, we have shown that the internal ultrastructures of individual *Ram*. *varieornatus* anhydrobionts are slightly different from those in active hydrated individuals, which contrasts with a previous report showing the neosynthesis of a specific extracellular structure associated with deep internal ultrastructural modifications in anhydrobiotic *Hys*. *exemplaris* individuals compared to hydrated individuals [70].

It is possible that the removal of the specific extracellular structure from desiccated *Hys*. *exemplaris* during the anhydrobiosis exit may slow the entire rehydration process, while *Ram*. *varieornatus*, lacking a detectable equivalent anhydrobiosis-specific ultrastructure, may not be influenced by the same way during the rehydration.

In summary, the desiccation process of *Ram*. *varieornatus* does not appear to be equivalent to that of *Hys*. *exemplaris*. These differences may at least partially explain the significant differences in desiccation resistance between both species.

## Supporting information

S1 Fig Distribution of mitochondrial size frequency depending on the tardigrade status. (TIF)
