# Supplementary figures and images for: A comparative ultrastructure study of the tardigrade Ramazzottius varieornatus in the hydrated state, after desiccation and during the process of rehydration
Source: PLoS One. 2024 Jun 6;19(6):e0302552. doi: 10.1371/journal.pone.0302552 (PMC11156355; doi:10.1371/journal.pone.0302552)

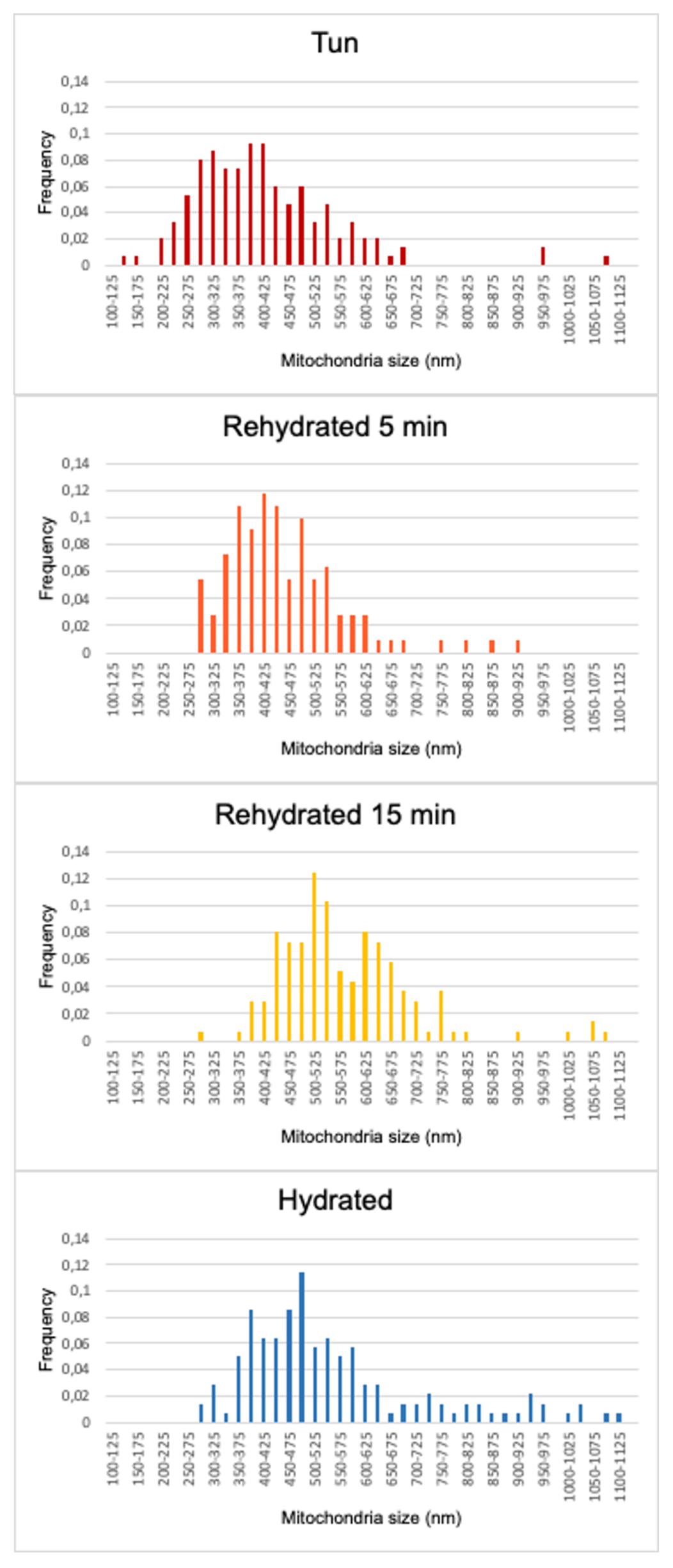

Supplement: S1 Fig — (TIF) [file pone.0302552.s001.tif]
